# Subgroup effects despite homogeneous heterogeneity test results

**DOI:** 10.1186/1471-2288-10-43

**Published:** 2010-05-17

**Authors:** Rolf HH Groenwold, Maroeska M Rovers, Jacobus Lubsen, Geert JMG van der Heijden

**Affiliations:** 1 Julius Center for Health Sciences and Primary Care, University Medical Center Utrecht, Utrecht, The Netherlands; 2 Department of Epidemiology and Biostatistics, Erasmus Medical Center Rotterdam, Rotterdam, The Netherlands

## Abstract

**Background:**

Statistical tests of heterogeneity are very popular in meta-analyses, as heterogeneity might indicate subgroup effects. Lack of demonstrable statistical heterogeneity, however, might obscure clinical heterogeneity, meaning clinically relevant subgroup effects.

**Methods:**

A qualitative, visual method to explore the potential for subgroup effects was provided by a modification of the forest plot, i.e., adding a vertical axis indicating the proportion of a subgroup variable in the individual trials. Such a plot was used to assess the potential for clinically relevant subgroup effects and was illustrated by a clinical example on the effects of antibiotics in children with acute otitis media.

**Results:**

Statistical tests did not indicate heterogeneity in the meta-analysis on the effects of amoxicillin on acute otitis media (Q = 3.29, p = 0.51; I^2 ^= 0%; T^2 ^= 0). Nevertheless, in a modified forest plot, in which the individual trials were ordered by the proportion of children with bilateral otitis, a clear relation between bilaterality and treatment effects was observed (which was also found in an individual patient data meta-analysis of the included trials: p-value for interaction 0.021).

**Conclusions:**

A modification of the forest plot, by including an additional (vertical) axis indicating the proportion of a certain subgroup variable, is a qualitative, visual, and easy-to-interpret method to explore potential subgroup effects in studies included in meta-analyses.

## Background

Practice guidelines increasingly rely on systematic reviews and meta-analyses. The ultimate purpose of a meta-analysis is to produce an overall estimate of the effect of an intervention by quantitatively combining study results. However, several issues arise in the process of integrating evidence. One of the main issues concerns heterogeneity, i.e. the extent to which different studies give similar or different results. Statistical tests are routinely available to evaluate the presence of statistical heterogeneity (between-study heterogeneity) in meta-analysis [1-3]. Strictly speaking, however, one is not really interested in statistical heterogeneity. What one is interested in is *clinical *heterogeneity, i.e., specific causes that underlie heterogeneity across studies, especially since the direction and magnitude of the effect in the meta-analysis is often used to guide decisions about clinical practice for a wide range of patients. Yet, relevant subgroup effects may not be revealed by a test for (statistical) heterogeneity. In meta-regression analysis the relation between a certain subgroup characteristic and the size of the treatment effect can in fact be quantified, but such analyses might be difficult to conduct or interpret, and rely on several assumptions. Furthermore, the observed treatment effect and subgroup variables are actually estimates, rather than true values. Ordinary meta-regression analysis (weighted least squares) does not take measurement errors in treatment and subgroup variables adequately into account and may consequently give a biased estimate of the slope of the regression line [4]. We will show that clinically relevant subgroup effects can be explored in a simple manner by modifying the forest plot.

## Methods

### Tests for heterogeneity

Several tests have been developed to assess heterogeneity. The so-called Cochrane's Q (or Cochrane's χ^2 ^test) weights the observed variation in treatment effects by the inverse of the variation in each study [5]. A large value of Q indicates large differences between studies, and hence, the effects from the included studies can be considered heterogeneous [2]. A modification of Cochrane's Q is the measure I^2^, which is the ratio of variation that exceeds chance variation and the total variation in the treatment effects. Possible values for I^2 ^range from zero to one, with a high value for I^2 ^indicating much heterogeneity. Both Q and I^2 ^are standardized measures, meaning that they don't depend on the metric of the effect size. A third measure of heterogeneity, indicating the variance of the true effect sizes is T^2^, where (similar to Q and I^2^) large values of T^2 ^indicate heterogeneity. This method of estimating the variance between studies (T^2^) is also known as the method of moments, or the DerSimonian and Laird method [6]. A fourth measure is the prediction interval, which indicates the distribution of true effect sizes and is based on T^2 ^[2]. Cochrane's Q is sensitive to the number of studies and especially when the number of studies included in a meta-analysis is small, Cochrane's Q too often leads to false-positive conclusions (too large type I error) [7]. The modification I^2 ^takes account of the number of included studies and has a correct probability of a type I error [3]. The measure T^2 ^is insensitive to the number of studies as well, but sensitive to the metric of the effect size [2].

Currently, I^2 ^appears to be used routinely in most published meta-analyses. Interestingly, the observed amount of heterogeneity depends on the effect measure that is considered in a meta-analysis: little heterogeneity when considering odds ratios implies large heterogeneity when considering risk differences and vice versa [8]. The reason for this is analogous to effect measure modification in a single study: if odds ratios are the same between strata (e.g., age categories) of a single study, risk differences are likely to differ between strata.

### Consequences of heterogeneity

Tests for heterogeneity indicate whether the variation in observed effects is either large or small. When heterogeneity is low (non-significant) for the chosen effect measure, variation between effects from different studies is (relatively) small. Thus, a fixed effects model can be used to synthesize the data, since the assumption underlying a fixed effects model is that the treatment effect is the same in each study, and variation between studies is due to sampling (i.e., chance) [3,7]. If variation in the effects found in the different studies is (relatively) large they could be considered as sampled from a distribution of effects, i.e., the true treatment effect that is estimated in the different studies is not a single value, but rather a distribution of effects. In that case, a random effects model has been recommended [3,7]. It has also been suggested that heterogeneity is inevitable in meta-analysis [9], and random effects models are therefore obligatory. If, however, heterogeneity is (very) large, one could even consider not pooling results from different studies at all, since studies are likely to be (very) different [2]. Furthermore, if there is a cause for heterogeneity, for example a subgroup effect, neither fixed nor random effects models take such relations between the effect size and subgroups into account. Another explanation for heterogeneity (other than differential treatment effects) could be a systematic error in the included studies. For example, systematic error that is related to e.g., the proportion of women, or differences in methodology (e.g., differences in outcome ascertainment) of the included studies [10].

### Relevant subgroup effects

**Figure 1:**
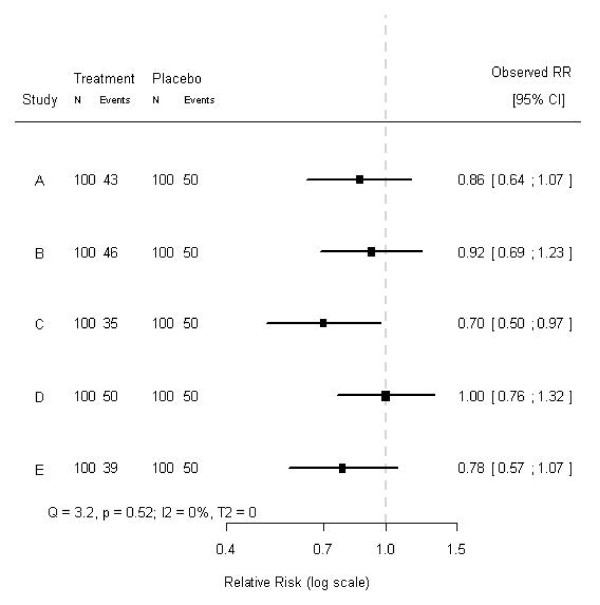
**Forest plot of a hypothetical meta-analysis of five studies**. The treatment effects and corresponding 95% confidence intervals of five studies included in a hypothetical meta-analysis are shown. The dashed line (- - - -) indicates no treatment effect. The studies are ordered chronologically.

**Figure 2:**
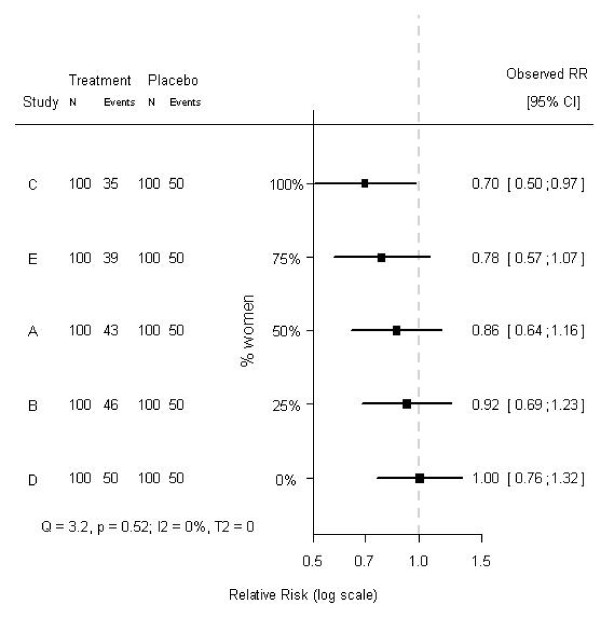
**Modified forest plot of a hypothetical meta-analysis of five studies, ordered by the proportion of women in each study**. The treatment effects and corresponding 95% confidence intervals of five studies included in a hypothetical meta-analysis are shown. The dashed line (- - - -) indicates no treatment effect. The studies are ordered by the proportion of women that was included in each study. The studies differ on the proportion of included women (0%, 25%, 50%, 75%, and 100%). The hypothetical treatment is effective in women (RR = 0.7), but not in men (RR = 1.0).

Test for heterogeneity do not indicate possible causes for heterogeneity. In fact, testing for heterogeneity in two meta-analyses - one with a clear cause for heterogeneity (e.g., a subgroup effect), and the other not - can lead to the same conclusions with respect to heterogeneity. For example, consider a hypothetical meta-analysis of five randomized trials on the effects of some treatment. Each trial consisted of 200 subjects, randomized to either treatment or placebo and the baseline risk for the outcome was 50%. The effects and their 95% confidence intervals are shown in Figure 1. Testing for heterogeneity indicated that these effects could not be considered heterogeneous (Q = 3.2, p = 0.52; I^2 ^= 0%; T^2 ^= 0). A closer look at the individual trials revealed that the proportion of women included in the studies differed considerably: from 0% to 100%. Rearranging the order of the effects by the proportion of women included in each study resulted in Figure 2. The tests for heterogeneity reached exactly the same conclusions, since the ordering of the observed treatment effects is not taken into account when testing for heterogeneity. Clearly, in the modified forest plot (Figure 2) the data have a certain pattern, which may indicate a differential treatment effect among men and women, i.e., modification of the treatment effect by sex. In fact, the treatment was effective in women (RR = 0.7), but not in men (RR = 1.0), and when analyzing the individual patient data (i.e., fitting a regression model to the individual patient data rather than the aggregated data and including a factor to account for differences between trials), a statistically significant subgroup effect was indeed found (p-value for interaction 0.011). Hence, in aggregated data the differential treatment effect by sex was not indicated by tests for heterogeneity, but only suggested by the modified forest plot, whereas in individual patient data this differential effect was clearly observed and statistically significant. Whether this subgroup effect is clinically relevant is rather subjective, but we can conclude that tests for heterogeneity on aggregated data appear not to tell the whole story about heterogeneity on individual patient data.

What is important is that a regular forest plot (Figure 1) only contains a horizontal axis (indicating the effect size), whereas the modified forest plot (Figure 2) contains two axes. Both in the regular forest plot and in the modified forest plot the horizontal axis indicates the effect size. The additional vertical axis in the modified forest plot indicates the proportion of a certain subgroup variable in the included studies. Importantly, the vertical axis does not simply indicate the order of the subgrouping variable, but also scales this variable.

An often used quantitative approach to investigate the association between a certain subgroup characteristic and the size of the treatment effect is by applying meta-regression analysis [11]. Such analyses however rely on several assumptions, e.g., linearity of the association, and might be hard to interpret for their quantitative nature. Furthermore, in ordinary meta-regression analysis the treatment effects from the included studies are handled as if they are true values rather than estimates, which can result in bias when using least squares regression [4]. In addition, aggregated data meta-(regression) analyses are inappropriate to estimate unbiased treatment effects in patient subgroups, since such comparisons are observational by nature. As a result, the observed subgroup effect may be attributable to other variables than the subgrouping variable [12]. Furthermore, as indicated before, neither fixed nor random effects models address the cause for heterogeneity. Individual patient data meta-analysis can be a valid alternative to study subgroup effects [12]. In conclusion, the modified forest plot is a qualitative, visual alternative to assess the potential for a clinical relevant subgroup effects.

## Results

**Figure 3:**
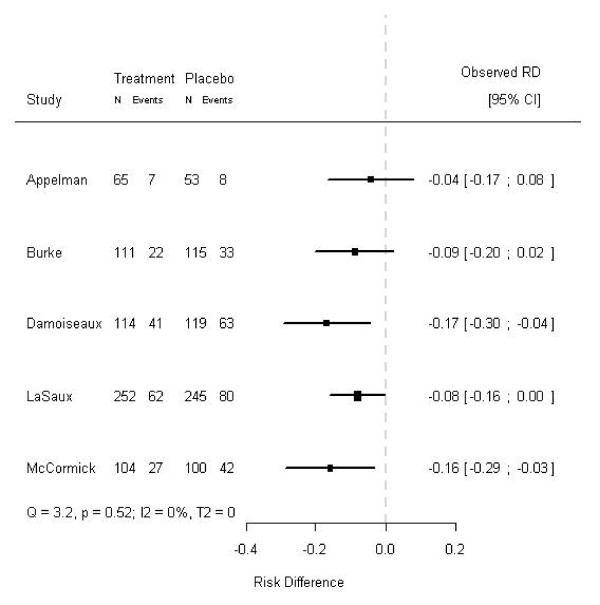
**Forest plot of a meta-analysis on five studies on the effects of amoxicillin in children with acute otitis media**. The treatment effects and corresponding 95% confidence intervals of five out of six randomized trials included in an IPD meta-analysis on the effects of amoxicillin in children with acute otitis media are shown [13]. The dashed line (- - - -) indicates no treatment effect. The studies are ordered chronologically. In the excluded study, bilaterality was not registered.

**Figure 4:**
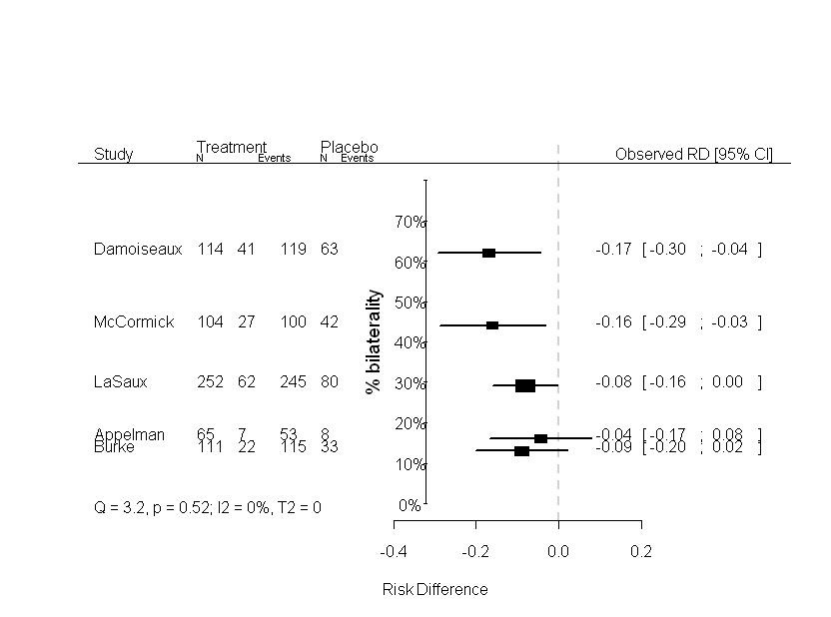
**Modified forest plot of a meta-analysis on five studies on the effects of amoxicillin in children with acute otitis media, ordered by the proportion of bilateral otitis in each study**. The treatment effects and corresponding 95% confidence intervals of five out of six randomized trials included in an IPD meta-analysis on the effects of amoxicillin in children with acute otitis media are shown [13]. The dashed line (- - - -) indicates no treatment effect. The studies are ordered by the proportion of children with bilateral otitis. In the excluded study, bilaterality was not registered.

In empirical meta-analyses subgroup effects can lead to patterns in a modified forest plot as well, as is illustrated by the following clinical example. In an individual patient data (IPD) meta-analysis on the effects of amoxicillin in children with acute otitis media, amoxicillin was more effective in children with bilateral otitis (p-value for interaction 0.021) [13]. Prior to this IPD meta-analysis age was thought to modify the effects of amoxicillin. Most of the studies included in the meta-analysis explicitly mentioned age distributions, but did not report the proportion of bilateral otitis. If the included studies, however, had reported the proportion of bilateral otitis, indications for the differential treatment effect found in the IPD meta-analysis could already have been suggested in a meta-analysis on the aggregated data (i.e., by constructing a modified forest plot). In Figure 3, the studies are chronologically ordered, whereas in Figure 4 their order is based on the proportion of children with bilateral acute otitis media (modified forest plot). As in the aforementioned example on the hypothetical meta-analysis, the measures of heterogeneity are the same for the Figures 3 and 4, as the order of the effects is not taken into account (Q = 3.29, p = 0.51; I^2 ^= 0%; T^2 ^= 0). What is striking, though, is the apparent relation between the proportion of bilaterality and the effects of amoxicillin in the different trials. Hence, based on the modified forest plot (Figure 4) a differential treatment effect for children with and without bilateral otitis is suggested. Similar to the hypothetical data presented above, in this clinical example tests for heterogeneity on aggregated data did not concur with the test for heterogeneity on individual patient data.

## Discussion and conclusions

Neither the absence nor the presence of heterogeneity (as indicated by the result of heterogeneity tests) in aggregated data meta-analyses appears to be indicative for subgroup effects. Irrespective of statistical test results, subgroup effects can be present in the data. A modified forest plot, including an additional (vertical) axis indicating the proportion of the subgroup variable (e.g., the proportion of bilateral otitis), may be helpful to identify clinically relevant subgroup effects. Unfortunately, patterns in a modified forest plot can only indicate (qualitative) subgroup effects. To quantify a subgroup effect, meta-regression analysis can be applied, but validity of results is then subject to several assumptions, and individual patient data meta-analysis might be needed [12]. The modified forest plot is a qualitative, visual, and easy-to-interpret alternative for exploring potential clinically relevant subgroup effects in studies included in aggregated data meta-analyses and should be considered when exploring such effects.

## Competing interests

The authors declare that they have no competing interests.

## Authors' contributions

All authors contributed to the conception and design of the study, analyses, and interpretation of data. RG drafted the manuscript, and all authors provided critical revision. RG had full access to all of the data in the study and takes responsibility for the integrity of the data and the accuracy of the data analysis. All authors read and approved the final manuscript.

## Pre-publication history

The pre-publication history for this paper can be accessed here:

http://www.biomedcentral.com/1471-2288/10/43/prepub
